# Research on the Characteristics of Infrared Radiation Spectra at Zigong Acupoint within Menstrual Cycle of Healthy Female

**DOI:** 10.1155/2019/9568324

**Published:** 2019-07-30

**Authors:** Meng Qin, Si-qi Ma, Zhi-ming Huang, Xue-yong Shen, Jian-zi Wei

**Affiliations:** ^1^School of Acupuncture and Massage, Shanghai University of Traditional Chinese Medicine, Shanghai 201203, China; ^2^Shanghai Institute of Technical Physics, Chinese Academy of Sciences, Shanghai 200083, China

## Abstract

The purpose of this study was to observe the characteristics of infrared radiation spectrum of Zigong acupoint (EX-CA1) within the menstrual cycle of healthy female. We used highly sensitive infrared radiation spectrum detection system and phase-locked amplification technology to detect and analyse the infrared radiation spectrum from 1.5*μ*m to 18*μ*m of 32 healthy female before, during, and after menstruation at EX-CA1 and control points. The results showed that the total radiation intensity of left EX-CA1 was significantly higher than that of left control point (*P* <0.05) at the whole menstrual cycle, and the difference between right EX-CA1 and right control points was statistically significant before and after menstruation (*P *<0.05), no statistical difference during menstruation. Previous studies found that the radiation near 15*μ*m was correlated with glucose metabolism. The results of this study showed that there were statistical differences in 10 wavelengths between left EX-CA1 and left control point from 14*μ*m to 18*μ*m, and there were statistical differences in 3 wavelengths on the right side (*P *<0.05). The left side is more prominent than the right side. The infrared radiation intensity of EX-CA1 decreased gradually with the change of cycle rhythm within menstrual cycle, but there was no statistical difference in this trend. There was no statistical difference in total radiation intensity between the right and left side of EX-CA1. Compared with the control points, the number of different wavelengths between left EX-CA1 and left control point during menstruation was significantly larger than that between right EX-CA1 and right control point (*P *<0.001). The results indicated that the energy of EX-CA1 was higher than control points. There was no difference in the radiation intensity between the right and left sides of EX-CA1 but there was acupoint laterality compared with nonacupoints. There was no significant rhythmic change in infrared radiation intensity of EX-CA1 during the menstrual cycle.

## 1. Introduction

There is complex interaction between the body surface and the internal organs. The diagnosis and treatment of visceral diseases by body surface abnormalities and the treatment of visceral diseases by body surface stimulation depend on the two-way relationship between body surface and viscera. Compared with other parts of the body surface, the relationship between acupuncture points and viscera is closer. The pathological responding points of the visceral functional abnormalities are mostly located at the corresponding acupoints, and the stimulation at the acupoints generally shows better therapeutic effects than the nonacupoint. Such difference between the acupoints and other parts of the body surface is also called acupoint function specificity which is the primary evidence of the existence of acupoints. Therefore, it has become the focus and hotspot of the basic research in acupuncture and moxibustion [1–3].

The functions of acupoints are closely related to the state of human functionality [4]. In the physiological state, the acupoints are often “Silenced” [5, 6] with little difference compared with the nonacupoint. The physiological state is also the basis for the study of acupoint function specificity with the same significance as the understanding of the “sensitization” characteristics of acupoints [7, 8] under pathological states. Therefore, it is especially important to identify the biophysical indicators that can sensitively distinguish the acupoints from the nonacupoint. Abundant physiological and pathological information can be found from the infrared spectra collected from the acupoints where stimulated radiation can also be tracked [9]. These types of insignificant physiological and pathological information are covered by the thermoinduced radiation and the techniques with more sensitive infrared detection capacity are required. We developed a highly sensitive detection system of the acupoint infrared radiation spectra with the mercury cadmium telluride infrared sensor and the lock-in amplification technology, which has been used in the preliminary studies of the spectra at the acupoints of the patients with heart diseases [10–12], pulmonary dysfunction [13, 14], and lobular hyperplasia [15], etc. However, there are few in-depth researches on the infrared spectra characteristics of acupoints under physiological conditions.

Menstrual cycle (MC) is the special physiological state in female of childbearing age, and the physiological functions change greatly during menstruation. Therefore, MC is quite suitable for the acupoint-specific research under physiological conditions. Previous studies [16, 17] have also found that the acupoints can respond sensitively to the changes in qi and blood during the MC. EX-CA1 is the extraordinary points with the indications of lower abdominal pain and blood stasis. It is a commonly used acupoint for treating gynecological diseases. This study is expected to observe the variation of infrared spectra at EX-CA1 within the menstruation cycle, so as to comprehensively understand the dynamic process of acupoint functionality, and to provide reference for the studies of acupoint “sensitization” and other specific studies of the acupoint functionality.

## 2. Materials and Methods

The subjects were 32 female college students, with an average age of 24±2 years, an average MC of 31±3 days, and an average duration of 6±1 days per MC. Inclusion criteria were [16] no abnormality in physical examination, no heart, lung, liver, kidney, or other medical diseases, no reproductive system diseases, normal MC, no dysmenorrhea, cold and other diseases during the test period.

The detection system of the acupoint infrared spectra was an upgraded version of the original detection system [11], which was jointly developed by the Shanghai Institute of Technical Physics, Chinese Academy of Sciences and Shanghai University of Traditional Chinese Medicine. The test range was from 1.5*μ*m to 18*μ*m, with 0.25*μ*m step for each band. The test points were the EX-CA1 and their lateral control points about 1 cm apart. According to the National Standard of the People's Republic of China (GBT 12346-2006) “Nomenclature and location of acupuncture points,” the EX-CA1 were located at 4 inches below the umbilicus and 3 inches left and right to the ventral midline. The detection methods and experimental conditions were of the same as those in the previous studies [11–13]. A total of 3 tests were performed on each subject, ovulation phase before menstruation period, 2 or 3 days after menstrual onset, and 1 or 2 days after menstruation period. The time of the day was kept the same for all tree tests for each subject to avoid the influence of circadian rhythm.

SPSS 21.0 software was used for statistical analysis, with *P*<0.05 considered as statistically significant, and paired t-test was used in the comparisons of infrared radiation intensity between the acupoints and the control points. Chi-square test was used to compare the number of wavelengths with differences between acupoints and control points. Variance analysis of mixed design was used to compare the infrared radiation intensity of acupoints and control points at different time points of menstrual cycle.

## 3. Results and Analysis

### 3.1. Comparison of Infrared Radiation between EX-CA1 and Control Points

#### 3.1.1. Characteristics of Infrared Radiation Spectra of EX-CA1 and Control Points

**Figure 1:**
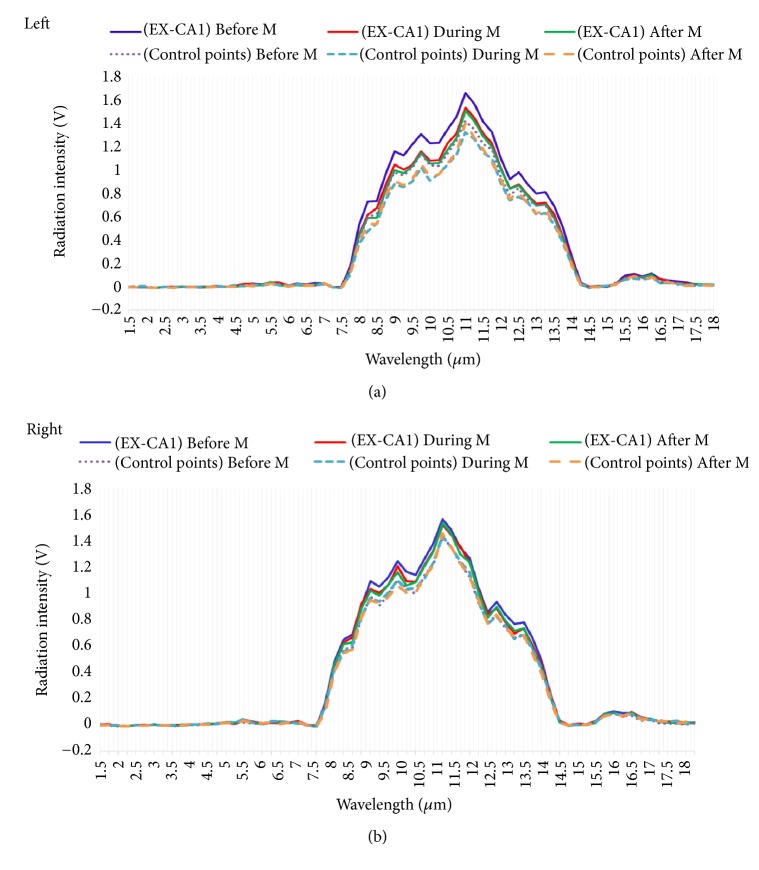
Comparison of infrared radiation spectra of EX-CA1 and control point at three different periods of menstrual cycle. Note:* Before M*: before menstruation;* During M*: during menstruation;* After M*: after menstruation. Wavelengths range from 1.5*μ*m to 18*μ*m.

The infrared radiation spectra of EX-CA1 and control points were basically the same, with the first wave peak appearing from 7.5*μ*m to 14.25*μ*m and the second wave peak appearing from 15.25*μ*m to 16.5*μ*m; the infrared radiation of acupoints and control points were mainly concentrated from 7.5*μ*m to 14.25*μ*m (Figure 1).

#### 3.1.2. Comparison of Total Radiation Intensity Obtained from EX-CA1 and Control Points

**Figure 2:**
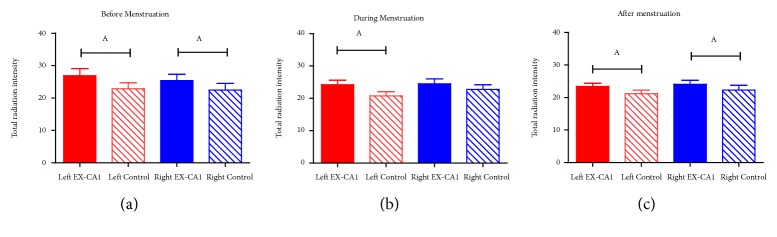
Comparison of the total radiation intensity obtained from EX-CA1 and control points at three different periods. Note: The total intensity is the sum of the intensity recorded of 67 wavelengths from 1.5*μ*m to 18*μ*m. ^A^ compared with control point,* P*<0.05.

The results of paired t-test showed that the total radiation intensity of right or left EX-CA1 was significantly larger than that of control points at all the three periods (*P*=0.004,* P*=0.0012,* P*=0.0018,* P*=0.0073,* P*=0.0205), except the comparison between the right EX-CA1 and right control point during menstruation (*P*=0.181) (Figure 2).

Our previous studies [10] have shown that, according to the Planck quantum energy formula En=nhv (n =0, 1, 2, 3…), the amount of heat emitted by the oxidation of a molecule of isocitric acid to alpha-ketoglutaric acid during sugar metabolism corresponds to a wavelength about 15*μ*m. Therefore, further analysis of the wavelength near the 15*μ*m wavelength related to glucose metabolism showed that there were significant differences in multiple wavelengths between EX-A1 and control points (Table 1). There were statistical differences in 10 wavelengths between left EX-A1 and left control points, among which 6 were before menstruation, 3 were during menstruation, and 1 was after menstruation. The corresponding infrared radiation intensity of left EX-A1 was significantly higher than that of left control points (*P*<0.05). There were statistical differences in 3 wavelengths between right EX-A1 and right control points, among which 2 were before menstruation and the corresponding infrared radiation intensity of right EX-A1 was significantly higher than that of right control points (*P*<0.05). 1 was after menstruation and the corresponding infrared radiation intensity of right EX-A1 was significantly lower than that of right control points

### 3.2. Variation of Infrared Radiation in EX-CA1 within Menstrual Cycle

**Figure 3:**
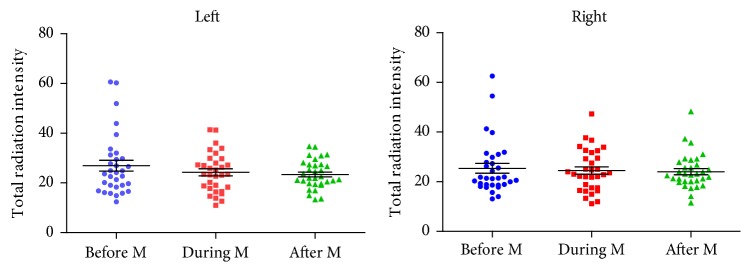
Variation of total infrared radiation intensity within menstrual cycle in EX-CA1. Note: Wavelengths range from 1.5*μ*m to 18*μ*m.

The total infrared radiation intensity of the left and right EX-CA1 showed the trend of before menstruation > after menstruation ≈ during menstruation. In order to determine whether there is still a correlation between the changes of infrared radiation intensity of uterus and menstrual cycle, a mixed design analysis of variance was conducted on the infrared radiation intensity of left and right EX-CA1 at three time points of menstrual cycle. The three different time points of menstrual cycle were used as within-subject factors, and the left and right acupoints were grouped as between-subject factors. Results show that time:* F *(1.821,112.911) =1.718,* P*=0.187, time & group:* F *(1.821,112.911) =0.368,* P*=0.673. It indicates that the infrared radiation intensity of the right and left EX-CA1 does not change significantly with the menstrual cycle. (Figure 3)

### 3.3. Comparison of Infrared Radiation of Bilateral EX-CA1

#### 3.3.1. Comparison of Infrared Radiation Intensity between Left and Right EX-CA1

There was no statistical significance in the total radiation intensity between the left and right side of EX-CA1 at three time points in the menstrual cycle (*P=0.3128,P=0.5365,P=0.8588*). Comparing the radiation intensity of 67 wavelengths (Table 2), the significant difference between the bilateral EX-CA1 was found at 2 wavelengths before menstruation (2.99%), 1 wavelength during menstruation period (1.49%), and 2 wavelengths after menstruation (2.99%) (*P*<0.05). The results indicated small difference between bilateral EX-CA1 at all three periods. It can be considered that the bilateral acupoints are equivalent, consistent with the previous research results [18].

#### 3.3.2. Comparison of Infrared Radiation Intensity between Each Side of EX-CA1 and Control Points

Although there was no significant difference in the total infrared radiation intensity between the bilateral EX-CA1, the acupoint specificity of the two sides was not completely consistent compared with the control points (Table 3(a)). Before and after menstruation, there were 31 and 23 different wavelengths between left EX-CA1 and control points and 25 and 26 different wavelengths between right EX-CA1 and control points, showing no statistically significant difference (P >0.05). The number of different wavelengths between left EX-CA1 and control points during menstrual period was 33, while the number on the right side was only 1, which was significantly larger on the left side than on the right side (P <0.001) (Table 3(b)).

## 4. Discussion

Infrared radiation is essentially a kind of energy. The higher infrared radiation intensity of EX-CA1 than that of control points indicates the higher energy radiated from the acupoints than from control points. Such results are consistent with those obtained by Hu Xianglong et al. who observed a higher intensity of the infrared radiation along the surface of meridians than that of the nonmeridian parts by using the infrared thermal imager [19, 20]. The high-energy radiation of human meridians and acupoints indicated stronger energy metabolism of tissues comparing with other nonmeridians or nonacupoints. The blood vessels at the acupoints are much denser than those at the nonacupoints [21]. It can explain the higher energy metabolism in meridians and acupoints. Our previous study also showed that the perfusion volume of Sanyinjiao (SP6) acupoint, which is more related to gynecological diseases, was significantly higher than that of the nonacupoint control points [22]. When the visceral lesions occur, the energy metabolism of the corresponding acupoints will also change, and the infrared radiation intensity of certain wavelength related to energy metabolism will also decrease [11, 12]. The in-depth study of the infrared radiation spectra of the acupoints can offer valuable references to clinical diagnosis. Thus, it is highly necessary to explore the infrared spectra characteristics of acupoints in physiological and pathological conditions.

The peak around 15*μ*m is closely related to glucose metabolism. The infrared radiation intensity of the wavelength related to glucose metabolism at acupoints was significantly higher than control points, suggesting that the glucose metabolism at acupoints was more vigorous than that at nonpoints. Clinically, the occurrence and development of various diseases may be closely related to the abnormalities of energy metabolism. Coronary heart disease [23, 24] has been identified as a metabolic disease, and a variety of gynecological diseases [25] have also been directly related to the abnormal glucose metabolism. The high energy metabolism and high glucose metabolism at the acupoints may be the etiology of the diseases applicable for acupoint treatment. The studies of the volt-ampere characteristics of the acupoints found the “energy storage” and “energy releasing” functions of the acupoints [26, 27]. The information exchange between the acupoints and organs is more fluent than the nonacupoints. Therefore, in-depth study is necessary to validate the underlying connection among the high-energy metabolism and high-glucose metabolism of acupoints, the “energy storage” and “energy releasing” functions of acupoints, and the fluent information exchange between the acupoints and organs.

The obvious fluctuation of Qi and blood in the body before menstruation and after menstruation indicated no corresponding response at EX-CA1 on the trunk. Previous studies have confirmed that the source acupoints along the six Yin Meridians located in the extremities can sensitively reflect the cyclical physiological changes such as circadian rhythm [27] and menstrual cycle [16]. In combination with the results of this study, further research is necessary to switch the targets from the trunk acupoints to the extremity acupoints in the studies of the acupoint functions with the periodic changes of the human body (such as monthly rhythm, day-night rhythm, seasonal rhythms, and others).

The acupoint functional difference between the bilateral acupoints with the same name is called acupoint laterality [28]. Wang Guangjun [29] found the functional laterality at Neiguan acupoints and also the Hegu acupoints with asymmetrical acupuncture performance [30]. The results of this study indicated that there was insignificant laterality of EX-CA1 before menstruation and after menstruation, but not during menstruation. This might be related to the physiological process of shedding uterine lining at the menstruation. Although the onset of menstruation is also a physiological process, the periodic bleeding caused by shedding uterine lining is also a damage to the uterus. The “EX-CA1” is closely related to the uterus, and the changes during menstruation could lead to the changes in the functions of EX-CA1. We proposed [31, 32] that, under pathological state, the acupoint responding areas or the meridian responding lines and bands may expand. Under such conditions, the original “nonacupoint” areas adjacent to the acupoints could become the “acupoint areas” and therefore, the difference between them might be lowered. The results of this experiment reverified this view. The responding area of the right EX-CA1 during menstruation became larger, and the original “nonacupoint” control point located 1 cm away from the acupoint was also covered in the EX-CA1 responding area, resulting in a smaller difference between the two points. If the left EX-CA1 has little such functional changes, the acupoint specificity might still exist. The different responses of the bilateral EX-CA1 to this physiological process of menstruation lead to the acupoint laterality at menstruation period. The experimental results also indicated that the relationships between right EX-CA1 and the shedding of uterine lining, bleeding during menstruation, were more significant than that in left EX-CA1.

## 5. Conclusion

Zigong (EX-CA1) acupoints of the healthy female have obvious acupoint specificity and indicated higher infrared radiation intensity and sugar metabolism than nonacupoint areas. The bilateral EX-CA1 of healthy female are basically equivalent; there is no difference in radiation intensity between the right and left sides of EX-CA1 but there is acupoint laterality comparing with nonacupoints. There is no significant rhythmic change in infrared radiation intensity of EX-CA1 during menstrual cycle. Right EX-CA1 is more related to the physiological changes in menstrual cycle, providing certain reference value for the selection of the acupoints in the treatment of the gynecological diseases with the acupuncture and moxibustion.

## Figures and Tables

**Table 1:** Comparison of the infrared radiation intensity at the relevant wavelength of glucose metabolism between EX-CA1 and control points $\left(\overset{-}{x}\pm s\right)$.

	Left			Right		
	Wavelength (*μ*m)	EX-CA1	Control point	Wavelength (*μ*m)	EX-CA1	Control point
Before Menstruation	14.25	0.036±0.090	0.018±0.062^a^	14.25	0.036±0.074	0.021±0.063 ^a^
	15.5	0.098±0.078	0.074±0.076 ^a^	16	0.091±0.084	0.070±0.080 ^a^
	16.25	0.114±0.104	0.083±0.077 ^a^			
	16.5	0.062±0.084	0.037±0.069 ^a^			
	17	0.044±0.076	0.017±0.042 ^a^			
	17.25	0.036±0.061	0.016±0.052 ^a^			

During Menstruation	15.5	0.081±0.056	0.058±0.049 ^a^			
	15.75	0.097±0.069	0.069±0.064 ^a^			
	16.5	0.067±0.063	0.030±0.057 ^a^			

After Menstruation	16.25	0.107±0.073	0.083±0.058 ^a^	17.25	0.017±0.056	0.029±0.058 ^a^

Note: This table only lists the data with significant statistical differences. Wavelengths range from 14.25*μ*m to 18*μ*m which is related to glucose metabolism. ^a^ compared with EX-CA1, *P*<0.05.

**Table 2:** List of wavelengths with statistical differences in infrared radiation intensity of bilateral EX-CA1 $\left(\overset{-}{x}\pm s\right)$.

	Wavelength (*μ*m )	Left EX-CA1	Right EX-CA1
Before Menstruation	6.75	0.031±0.049	0.016±0.036 ^a^
	14.75	0.003±0.026	0.008±0.031 ^a^
During Menstruation	4.75	0.023±0.035	0.011±0.024 ^a^
After Menstruation	6	0.007±0.033	0.010±0.030 ^a^
	6.5	0.007±0.032	0.024±0.048 ^a^

Note: Wavelengths range from 1.5*μ*m to 18*μ*m. ^a^ compared with Left EX-CA1, *P*<0.05.

**Table:** (a) The wavelength number of infrared radiation intensity with statistical differences between EX-CA1 and control points

	No. of Wavelengths (The infrared radiation intensity of acupoints> that of control points)		No. of Wavelengths (The infrared radiation intensity of acupoints< that of control points		Total No. of Wavelengths with statistical differences between EX-CA1 and control points	
	Left	Right	Left	Right	Left	Right

Before Menstruation	31	25	0	0	31	25
During Menstruation	33	1	0	0	33	1
After Menstruation	23	25	0	1	23	26

Note: Wavelengths range from 1.5*μ*m to 18*μ*m.

**Table:** (b) Chi-square tests of the wavelengths number with statistical differences in infrared radiation intensity between EX-CA1 and control points

		No. of Wavelengths without statistical difference	No. of Wavelengths with statistical difference	x^2^-value	P-value
Before Menstruation	Left	35	31	1.272	=0.341
	Right	42	25		

During Menstruation	Left	34	33	40.358	<0.001
	Right	66	1		

After Menstruation	Left	43	23	0.224	=0.769
	Right	41	26		

## Data Availability

The relevant raw data can be obtained by contacting the first author or corresponding author.
